# Detection of leukemia gene fusions on DNA-level through targeted Next-Generation Sequencing

**DOI:** 10.1371/journal.pone.0332407

**Published:** 2025-10-09

**Authors:** Bin Chen, Zhihui Gao, Long Chen, Hong Zhang, Yani Lin, Jing Li

**Affiliations:** 1 Sino-US Diagnostics Lab, Tianjin Enterprise Key Laboratory of AI-aided Hematopathology Diagnosis, Tianjin, China; 2 Department of Pathology, Zibo Central Hospital, Zibo, Shandong, China; Hirosaki University Graduate School of Medicine, JAPAN

## Abstract

**Background:**

Targeted Next-Generation Sequencing (tNGS) is commonly used to detect genetic mutations in patients with leukemia on DNA-level, necessitating additional methods to confirm genomic rearrangements and gene fusions, resulting in a substantial sample volume requirement and significant labor expenses.

**Methods:**

A novel custom leukemia tNGS panel, independently developed by Sino-US Diagnostics Lab, includes all exons from 302 genes closely associated with leukemia and 95 introns from 26 genes, thereby facilitating the detection of gene fusion alterations on DNA-level. Additionally, the common breakpoint regions of *IGH* and *MYC* are employed for the detection of *IGH* or *MYC* rearrangements. Commercial quantitative reverse transcription polymerase chain reaction (qRT-PCR) reagents were used simultaneously to detect 45 gene fusions in RNA samples. A total of 357 adults diagnosed with leukemia were included in the study.

**Results:**

The qRT-PCR method detected a total of 102 gene fusions, encompassing 23 distinct types. The tNGS method identified the same gene fusions on DNA-level in 98 samples, achieving a Positive Percent Agreement (PPA) of 96.1% (98/102) when compared to the qRT-PCR method, and no false positive findings. Additionally, it revealed the presence of two gene fusions, *KMT2A*::*ELL* and *KMT2A*::*MLLT3*, which had gone undetected by qRT-PCR. The tNGS can also identify *IGH* or *MYC* gene rearrangements in patients with B-ALL, achieving a PPA of 93.8% (15/16) when compared to the FISH. Moreover, tNGS can accurately identify the specific partner genes associated with these rearrangements, facilitating a more precise analysis of the impact of mutations on prognosis.

**Conclusion:**

This study confirms the feasibility of employing tNGS methods to concurrently identify gene mutations and fusions (including *IGH* and *MYC* rearrangements) at the DNA level in adults with leukemia.

## Introduction

Hematological malignancies encompass a range of conditions that impact the blood or immune system and stem from either the myeloid or lymphoid lineage. Genomic rearrangements are crucial somatic molecular aberrations that play essential roles in the initiation and advancement of hematologic malignancies. These genomic rearrangements have the potential to create gene fusions, disrupting the regulation of cell division and proliferation [1]. Gene fusions are pivotal markers for the diagnosis, selection of therapy, and prognosis prediction through risk stratification in hematologic malignancies [2,3]. In the 5th edition of the World Health Organization Classification of Haematolymphoid Tumours (WHO-HAEM5) and the 2022 international consensus classification (ICC) [4], the diagnosis of acute myeloid leukemia (AML) and acute lymphoblastic leukemia (ALL) necessitates the identification of multiple defining genetic abnormalities, primarily various gene fusions [5,6].

The commonly used methods for detecting genomic rearrangements or gene fusions currently include: chromosome banding analysis (CBA), fluorescent *in situ* hybridization (FISH), quantitative reverse transcription polymerase chain reaction (qRT-PCR) and RNA sequencing (RNA-seq). Karyotyping is still the most commonly used and unbiased method for assessing chromosomal abnormalities, its main limitations include the need for live culturable cells, complex experimental procedures, low resolution, and low sensitivity [7]. FISH and qRT-PCR are also very commonly used methods for detecting chromosomal abnormalities, both with high sensitivity and short turnaround times. FISH and qRT-PCR are limited to the identification of genetic alterations in specific target regions and the recognition of specific gene fusions, posing challenges when applied to the comprehensive screening of numerous gene fusions [8,9]. RNA-seq enables the simultaneous detection of hundreds or even thousands of gene fusions [10,11], offering an advantageous method for comprehensive fusion gene screening in patients for disease diagnosis. Nonetheless, its high cost poses a barrier to its extensive utilization. *IGH*-related rearrangements, including *IGH*::*MYC*, *IGH*::*CRLF2*, and *IGH*::*IL3*, play crucial roles in the diagnosis and classification of B-ALL as well as in treatment guidance [3]. However, most of these rearrangements are unable to generate fusion gene transcripts at the RNA level [12,13], making qRT-PCR and RNA-seq methods inadequate for detecting such alterations.

Testing for gene mutations and gene fusions is pivotal in the diagnosis and treatment of leukemia [3]. Targeted Next-Generation Sequencing (tNGS) is commonly employed to identify mutations in DNA samples, while qRT-PCR is a widely utilized in molecular biology for detecting gene fusion events in RNA samples. This necessitates the use of multiple samples for multiple testing procedures, resulting in a substantial sample volume requirement and significant labor expenses. Utilizing tNGS to concurrently identify gene mutations and gene fusions in DNA has been demonstrated as feasible in solid tumors [14,15]. Can adult leukemia patients also use tNGS to simultaneously detect gene mutations and gene fusions in DNA to reduce the requirements for samples and experimental operations? Large-scale validation of this approach has not been undertaken yet.

## Materials and methods

### Patients

From May 2022 to July 2023, a total of 357 consecutive adult patients underwent tNGS testing at SINO-US Diagnosis Center. The study methodologies adhered to the standards outlined in the Declaration of Helsinki. Prior to analysis, all patient data underwent anonymization and de-identification procedures. The data supporting the findings of this study are available on Figshare at https://doi.org/10.6084/m9.figshare.26470501 and on the Sequence Read Archive (SRA) under bioproject_accession number PRJNA1263052. The dates on which the data were accessed for research purposes are 16/5/2025. Of the total cases, 241 were diagnosed with Acute Myeloid Leukemia (AML), including 18 cases of Acute Promyelocytic Leukemia (APL); 88 cases were identified as Acute Lymphoblastic Leukemia (ALL), of these patients, 76 cases were B-cell ALL (B-ALL), and 12 cases were T-cell ALL (T-ALL). Furthermore, 28 cases were classified as Chronic Myeloid Leukemia (CML).

### The design of the leukemia tNGS panel for the detection

A novel custom leukemia tNGS panel, independently developed by Sino-US Diagnostics Lab, aims to simultaneously detect gene mutations and gene fusions at the DNA level. The panel comprises all exons of 302 genes closely associated with leukemia (S1 Table), enabling the detection of single nucleotide variants (SNVs) and small insertions and deletions (indels) within the coding regions of these genes. Additionally, it incorporates 94 introns from 26 genes for the detection of gene fusion alterations within these regions (Table 1). The selection of these introns was guided by the documented gene breakpoint positions reported in published articles. According to the characteristics of tNGS testing, gene fusions can be captured by partial matching with the detection probes. Subsequent tNGS sequencing can reveal the specific genes involved in the fusion event. Thus, the testing panel only needs to cover one end of the fusion gene, theoretically enabling the detection of fusion with any other gene. For example, this detection panel only includes the introns of the *NUP98* gene, but it can simultaneously detect various fusion types such as *NUP98*::*NSD1*, *NUP98*::*HOXA11*, *NUP98*::*HOXA13*, *NUP98*::*HOXA9*, and so on. For gene fusions with higher occurrence rates in leukemia, such as *BCR*::*ABL1*, *CBFB*::*MYH11*, *PML*::*RARA*, this panel includes both ends of the gene fusions, hoping to increase the detection rate of these critical gene fusions. Simultaneously, we referenced the common breakpoint regions of *IGH* and *MYC* identified in lymphoma studies [16–18] and integrated these regions into our panel detection (Table 1). The detection of fusion in these genes fulfills the diagnostic criteria for AML and B-ALL, as defined by genetic abnormalities in the 5th edition of the World Health Organization Classification of Haematolymphoid Tumours (WHO-HAEM5). The total length of all introns, as well as the rearrangement regions of the *IGH* and *MYC* genes included in this study, is 679.9 kilobase pairs (Kb).

**Table 1:** Details of the genes and their target regions included in the design of the leukemia tNGS panel to analyze translocations in DNA samples.

Gene	Transcript ID	Intron regions	Reference
ABL1	NM_007313.3	intron 1 ~ 3	[19,20]
BCR	NM_004327.4	intron 6, 13, 14, 19	[19,20]
CBFB	NM_022845.3	intron 4 ~ 6	[20]
ETV6	NM_001987.5	intron 4 ~ 7	[20]
FUS	NM_004960.4	intron 5 ~ 8	[21]
HLF	NM_002126.5	intron 3	[22,23]
JAK2	NM_004972.4	intron 6, 8 ~ 11, 14, 16, 18	[24]
KMT2A	NM_001197104.2	intron 7 ~ 12, 21 ~ 23	[25]
MEF2D	NM_005920.4	intron 4 ~ 9	[26]
MLLT3	NM_004529.4	intron 3, 7, 8	[27]
MYH11	NM_022844.3	intron 6, 7, 11	[20]
NPM1	NM_002520.7	intron 3, 4, 6, 8	[28]
NUP214	NM_005085.4	intron 5, 16 ~ 18, 31 ~ 34	[29,30]
NUP98	NM_005387.7	intron 11 ~ 16	[31]
PBX1	NM_002585.4	intron 1, 4	[20]
PDGFRA	NM_006206.6	intron 11, 12	[32]
PDGFRB	NM_002609.4	intron 8 ~ 11	[32]
PICALM	NM_007166.4	intron 17 ~ 19	[33,34]
PML	NM_002675.4	intron 3, 6	[20]
RARA	NM_000964.4	intron 2	[20]
RUNX1	NM_001754.5	intron 1, 5, 6	[20]
STIL	NM_003035.2	intron 1	[20]
TAL1	NM_003189.5	intron 2	[20]
TCF3	NM_003200.5	intron 11, 13, 15 ~ 17	[22,23]
ZNF384	NM_133476.5	intron 1, 3, 7	[35]
RBM15	NM_022768.5	intron 1	[36]
MYC	NM_002467.6	5’UTR, 5’ upstream 10 kb, intron 1 ~ 2, 3’UTR, 3’ downstream 10 kb	[16–18]
IgH	/	chr14:106024796–106044796; chr14:106156687–106176687 chr14:106239409–106242027; chr14:106310062–106385377 chr14:106329109–106331668; chr14:106471245–106471550 chr14:106494134–106494445; chr14:106518399–106518704 chr14:106725200–106725505; chr14:106815721–106816026 chr14:106829593–106829895; chr14:106877618–106877926 chr14:107034728–107035033; chr14:107169930–107170235 chr14:107178819–107179130; chr14:107329540–107349540	[16–18]

The research utilized the xGen™ Custom Hybridization Capture library application with capture probes provided by Integrated DNA Technologies (IDT, Coralville, IA). The panel was designed and optimized on the xGen™ platform, incorporating 6,534 specific 120-base pair (bp) probes to cover 95.1% (653.4 Kb) of the target intron region effectively. The relatively low intron coverage can be attributed to the high repetition of intron regions in the genome. Designing capture probes in these areas would lead to a waste of the final sequencing data.

### DNA/RNA extraction and tNGS sequencing

The DNA and RNA were concurrently extracted from bone marrow (BM) or peripheral blood (PB) by commercial kits (Tiangen Biochemical Technology Co., Ltd., Beijing, China). Transform DNA into libraries using commercial library construction kits (Kapa Biosystems, Wilmington, MA). The library was captured and enriched using the leukemia tNGS panel, and subsequently sequenced (PE150) on the Illumina NextSeq550 system. The mean coverage depth was ~ 1,000X per sample. After deduplication, an average sequencing read depth of over 400x is considered as quality control pass. The raw sequencing signal files were converted into FASTQ files using the default parameters of the bcl2fastq program. Subsequently, the FASTQ files underwent quality control through the default settings of the fastp software, which filtered out low-quality bases, uncertain bases, and reads containing adapters, thereby generating clean data in FASTQ files. The clean data that passed quality control were aligned to the human hg19 reference genome using the default parameters of the BWA-MEN software, resulting in BAM files. The Sambamba software was employed to remove duplicate reads from the BAM files. Variants were identified in the BAM files using VarDict software, producing VCF files. Finally, ANNOVAR software was utilized to annotate the variants with reference to relevant databases, including RefGene, dbSNP, ExAC, ESP6500, 1000 Genomes, COSMIC, and an in-house database. To detect gene fusions, we employed two software tools, Factera [37] and GeneFuse [38], for simultaneous analysis, with both utilizing default parameters. The reference file “cancer.hg19.csv” in GeneFuse was updated to include the coordinates of frequently occurring gene fusions in leukemia. A fusion gene is deemed authentic only if it is supported by six or more unique-reads (junction or spanning reads). A fusion gene was deemed successfully identified if detected by either fusion caller and supported by six or more unique-reads (junction or spanning reads).

### Detect 45 leukemia-related gene fusions using the qRT-PCR method

The extracted RNA was reverse-transcribed into complementary DNA (cDNA) using commercially available kits (Invitrogen SuperScript IV kit, Invitrogen, Carlsbad, CA). A screening of 45 gene fusions associated with leukemia was performed using an qRT-PCR kit (Zeesan Biotech, Xiamen, Fujian Province, China). Among the 45 gene fusions is *PML*::*RARA*, *RUNX1*::*RUNX1T1*, *CBFB*::*MYH11*, *BCR*::*ABL1*, *DEK*::*NUP214*, *FIP1L1*::*PDGFRA*, *FUS*::*ERG*, *KMT2A*::*MLLT3*, and so on, with a comprehensive list of all gene fusions and types provided in S2 Table. A qRT-PCR cycle threshold (Ct) value less than 30 for the target transcript is indicative of a positive result for the gene fusion. In order to correct variations in RNA quality and quantity and to calculate the sensitivity of each measurement, the control gene transcript should be amplified in parallel to the gene fusion transcripts. Every test includes the *ABL1* as the internal control gene, and the reliability of the test result is determined only when the Ct value of the ABL1 transcript is below 25.

### Cytogenetic analysis and FISH

The bone marrow cells were cultured for a duration of 24 h prior to the preparation of chromosomes. Subsequently, a total of 20 metaphase chromosomes were analyzed using R-banding techniques facilitated by Innovative Solutions for Automated Imaging (MetaSystem, Germany). All samples diagnosed with B-ALL were scheduled to undergo to FISH for detecting *IGH* (14q32) gene breakpoints aimed at analyzing rearrangements in the *IGH* gene. FISH was performed by using commercially available *IGH* dual-color break-apart rearrangement probes (Anbiping, Guangzhou, China).

### Ethics approval statement and patient consent statement

This study is designed as a retrospective observational analysis. All patients provided verbal consent for the use of NGS test results in scientific research. This consent procedure and the study protocol was reviewed and approved simultaneously by Medical Research Ethics Committee of SINO-US Diagnosis Center, Tianjing, China (approval number 202401, date of decision May 6, 2024).

## Results

### Comparison of qualification rates between tNGS and qRT-PCR

The DNA extracted from all 357 cases was subjected to tNGS analysis, with all sequencing data meeting the required quality control standards. Although some samples required repeated testing, they all achieved a mean sequencing read depth exceeding 400x after deduplication. After reverse transcription and qRT-PCR analysis of the RNA samples, a total of 12 samples, despite undergoing multiple rounds of testing, consistently showed inadequate amplification of the internal reference *ABL1* gene, with all Ct values failing to drop below 25. Consequently, these 12 samples were deemed unsuccessful in the analysis. The final success rate of qRT-PCR detection was determined to be 96.6% (345/357). The failure of these samples resulted from extended sample transportation duration and unsuitable storage conditions. The success rate of tNGS testing with DNA is higher than that of qRT-PCR testing with RNA (100% vs. 96.6%).

### Sequencing results of the intron regions

After sequencing and mapping, achieving an adequate sequencing depth in the target region is essential for identifying all variations. Insufficient sequencing depth in the target region can decrease the sensitivity of detecting variations in that area. Through the analysis of the sequencing depth of individual introns, particular regions demonstrating low sequencing depth were pinpointed. These regions may exhibit diminished probe binding affinity due to abnormal GC content, leading to reduced capture efficiency and consequently a decrease in sequencing depth. Low sequencing depth regions are defined as regions with sequencing depths below 20% of the sample’s average sequencing depth, in samples with an average sequencing depth of 1000x, it implies that the sequencing depth of these regions is below 200x. A total region of 55.0 Kb was identified as having low sequencing depth, representing 10.8% (55/506.7) of all intron regions, refer to S3 Table for specific chromosome coordinates. Locating the breakpoint within these regions can pose challenges in detecting the gene fusions, thereby impacting its positive detection rate. Several introns exhibit low sequencing depth, comprising over 40% of the total intron length. These introns include intron 8 of *KMT2A*, intron 8 of *NPM1*, intron 17 of *NUP214*, and intron 10 of *NUP98*.

### Consistency analysis of gene fusions detected by tNGS and qRT-PCR

**Fig 1:**
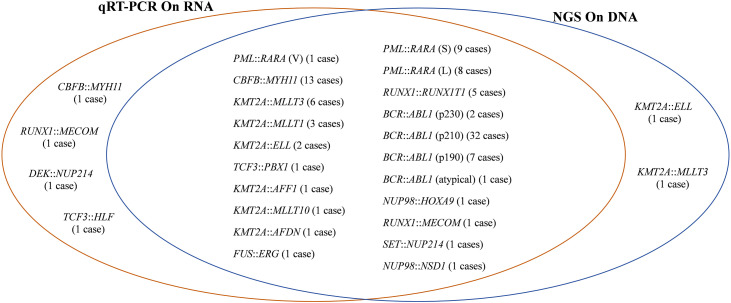
Gene fusions detected by qRT-PCR and tNGS. qRT-PCR identified a total of 102 gene fusions on RNA-level, whereas tNGS detected 98 out of the 102 gene fusions on DNA-level, leaving 4 gene fusions undetected at this level. Moreover, tNGS on DNA-level revealed two additional gene fusions not detected by qRT-PCR.

Analysis using qRT-PCR identified gene fusions in a total of 102 samples, with an overall positivity rate of 29.6% (102/345). Specifically, the samples exhibited 7 cases of *BCR*::*ABL1* (p190-type), 32 cases of *BCR*::*ABL1* (p210-type), 2 cases of *BCR*::*ABL1* (p230-type), 1 cases of atypical *BCR*::*ABL1* (e1a3), 14 cases of *CBFB*::*MYH11*, 9 cases of *PML*::*RARA* (S-type), 8 cases of *PML*::*RARA* (L-type), 1 case of *PML*:*RARA* (V-type), 6 cases of *KMT2A*::*MLLT3*, 5 cases of *RUNX1*::*RUNX1T1*, 3 cases of *KMT2A*::*MLLT1*, 2 cases of *KMT2A*::*ELL*, and 2 cases of *RUNX1*::*MECOM*. Additionally, each of the following gene fusions was detected once: *DEK*::*NUP214*, *SET*::*NUP214*, *FUS*::*ERG*, *KMT2A*::*AFDN*, *KMT2A*::*AFF1*, *KMT2A*::*MLLT10*, *NUP98*::*HOXA9*, *NUP98*::*NSD1*, *TCF3*::*HLF*, and *TCF3*::*PBX1* (Fig 1, Table 2, The detailed results can also be seen in S4 Table).

**Table 2:** The similarities and differences between the fusion gene results detected by qRT-PCR and tNGS methods.

Sample number	qRT-PCR detection result	tNGS detection result
AC529	*BCR*::*ABL1* (p190-type)	*BCR* (intron.1)::*ABL1* (exon.2)
AP1254	*BCR*::*ABL1* (p190-type)	*BCR* (intron.1)::*ABL1* (intron.1); *ABL1* (intron.1)::*BCR* (intron.1)
AP1344	*BCR*::*ABL1* (p190-type)	*BCR* (intron.1)::*ABL1* (intron.1)
AP1352	*BCR*::*ABL1* (p190-type)	*BCR* (intron.1)::*ABL1* (intron.1); *ABL1* (intron.1)::*BCR* (intron.1)
AP1376	*BCR*::*ABL1* (p190-type)	*BCR* (intron.1)::*ABL1* (intron.1)
AR425	*BCR*::*ABL1* (p190-type)	*BCR* (intron.1)::*ABL1* (intron.1)
AR564	*BCR*::*ABL1* (p190-type)	*BCR* (intron.1)::*ABL1* (intron.1); *ABL1* (intron.1)::*BCR* (intron.1)
AP405	*BCR*::*ABL1* (p210-type)	*BCR* (intron.13)::*ABL1* (intron.1)
AP410	*BCR*::*ABL1* (p210-type)	*BCR* (intron.14)::*ABL1* (intron.1)
AP415	*BCR*::*ABL1* (p210-type)	*BCR* (intron.14)::*ABL1* (intron.1); *ABL1* (intron.1)::*BCR* (intron.14)
AP426	*BCR*::*ABL1* (p210-type)	*BCR* (intron.14)::*ABL1* (intron.1)
AP486	*BCR*::*ABL1* (p210-type)	*BCR* (intron.14)::*ABL1* (intron.1); *ABL1* (intron.1)::*BCR* (intron.14)
AP496	*BCR*::*ABL1* (p210-type)	*BCR* (intron.14)::*ABL1* (intergenic)
AP502	*BCR*::*ABL1* (p210-type)	*BCR* (intron.13)::*ABL1* (intron.1)
AP535	*BCR*::*ABL1* (p210-type)	*BCR* (intron.14)::*ABL1* (intron.1); *ABL1* (intron.1)::*BCR* (intron.14)
AP536	*BCR*::*ABL1* (p210-type)	*BCR* (exon.14)::*ABL1* (intron.1); *ABL1* (intron.1)::*BCR* (intron.14)
AP546	*BCR*::*ABL1* (p210-type)	*BCR* (intron.13)::*ABL1* (intergenic); *ABL1* (intergenic)::*BCR* (intron.13)
AP566	*BCR*::*ABL1* (p210-type)	*BCR* (intron.14)::*ABL1* (intron.1); *ABL1* (intron.1)::*BCR* (intron.14)
AP576	*BCR*::*ABL1* (p210-type)	*BCR* (intron.13)::*ABL1* (intergenic); *ABL1* (intergenic)::*BCR* (intron.13)
AP584	*BCR*::*ABL1* (p210-type)	*BCR* (intron.14)::ABLl (intron.1)
AP596	*BCR*::*ABL1* (p210-type)	*BCR* (intron.14)::ABLl (intron.1); ABLl (intron.1)::*BCR* (intron.14)
AP628	*BCR*::*ABL1* (p210-type)	*BCR* (intron.14)::*ABL1* (intron.1); *ABL1* (intron.1)::*BCR* (intron.14)
AP633	*BCR*::*ABL1* (p210-type)	*BCR* (intron.14)::*ABL1* (intron.1); *ABL1* (intron.1)::*BCR* (intron.14)
AP638	*BCR*::*ABL1* (p210-type)	*BCR* (intron.13)::*ABL1* (intron.1)
AP665	*BCR*::*ABL1* (p210-type)	*BCR* (intron.14)::*ABL1* (intron.1)
AP667	*BCR*::*ABL1* (p210-type)	*BCR* (intron.14)::*ABL1* (intron.1)
AP668	*BCR*::*ABL1* (p210-type)	*ABL1* (intron.1)::*BCR* (intron.14)
AP680	*BCR*::*ABL1* (p210-type)	*BCR* (intron.14)::*ABL1* (intron.1)
AP681	*BCR*::*ABL1* (p210-type)	*BCR* (intron.14)::*ABL1* (intron.1)
AP689	*BCR*::*ABL1* (p210-type)	*BCR* (intron.13)::*ABL1* (intron.1)
AP751	*BCR*::*ABL1* (p210-type)	*BCR* (intron.14)::*ABL1* (intron.1)
AP753	*BCR*::*ABL1* (p210-type)	*BCR* (intron.13)::*ABL1* (intron.1); *ABL1* (intron.1)::*BCR* (intron.13)
AP762	*BCR*::*ABL1* (p210-type)	*BCR* (intron.14)::*ABL1* (intron.1); *ABL1* (intron.1)::*BCR* (intron.14)
AP763	*BCR*::*ABL1* (p210-type)	*BCR* (intron.13)::*ABL1* (intergenic)
AP765	*BCR*::*ABL1* (p210-type)	*BCR* (intron.14)::*ABL1* (intron.1)
AP830	*BCR*::*ABL1* (p210-type)	*BCR* (intron.13)::*ABL1* (intron.1); *ABL1* (intron.1)::*BCR* (intron.13)
AP869	*BCR*::*ABL1* (p210-type)	*BCR* (intron.14)::*ABL1* (intron.1)
AP903	*BCR*::*ABL1* (p210-type)	*BCR* (intron.13)::*ABL1* (intron.1); *ABL1* (intron.1)::*BCR* (intron.13)
AP904	*BCR*::*ABL1* (p210-type)	*BCR* (exon.15)::*ABL1* (intron.1); *ABL1* (intron.1)::*BCR* (exon.15)
AP1166	*BCR*::*ABL1* (p230-type)	*BCR* (intron19)::*ABL1* (intron1); *ABL1* (intron1)::*BCR* (intron19)
AP698	*BCR*::*ABL1* (p230-type)	*BCR* (intron19)-*ABL1* (intron1)
AP739	*BCR*::*ABL1* (atypical)	*BCR* (intron.1)::*ABL1* (intron.2); *ABL1* (intron.2)::*BCR* (intron.1)
AP420	CBFB::MYH11	*CBFB* (intron.5)::*MYH11* (intron.32)
AP479	CBFB::MYH11	*MYH11* (intron.32)::*CBFB* (intron.5)
AP488	CBFB::MYH11	*CBFB* (intron.5)::*MYH11* (intron.32); *MYH11* (intron.32)::*CBFB* (intron.5)
AP538	CBFB::MYH11	*CBFB* (intron.5)::*MYH11* (intron.32); *MYH11* (intron.32)::*CBFB* (intron.5)
AP541	CBFB::MYH11	*CBFB* (intron.5)::*MYH11* (exon.32); *MYH11* (exon.32)::*CBFB* (intron.5)
AP542	CBFB::MYH11	*CBFB* (intron.5)::*MYH11* (intron.32); *MYH11* (intron.32)::*CBFB* (intron.5)
AP624	CBFB::MYH11	*CBFB* (intron.5)::*MYH11* (intron.8); *MYH11* (intron.8)::*CBFB* (intron.5)
AP647	CBFB::MYH11	*CBFB* (intron.5)::*MYH11* (intron.32)
AP717	CBFB::MYH11	*CBFB* (intron.5)::*MYH11* (intron.32); *MYH11* (intron.32)::*CBFB* (intron.5)
AP734	CBFB::MYH11	*CBFB* (intron.5)::*MYH11* (intron.32); *MYH11* (intron.32)::*CBFB* (intron.5)
AP742	CBFB::MYH11	*CBFB* (intron.4)::*MYH11* (exon.26); *MYH11* (exon.26)::*CBFB* (intron.4)
AP798	CBFB::MYH11	*CBFB* (intron.5)::*MYH11* (intron.32); *MYH11* (intron.32)::*CBFB* (intron.5)
AP834	CBFB::MYH11	*CBFB* (intron.5)::*MYH11* (intron.32); *MYH11* (intron.32)::*CBFB* (intron.5)
AP484	CBFB::MYH11	Negative
AP458	RUNX1::RUNX1T1	*RUNX1* (intron.5)::*RUNX1T1* (intron.1)
AP708	RUNX1::RUNX1T1	*RUNX1* (intron.6)::*RUNX1T1* (intron.1); *RUNX1T1* (intron.1)::*RUNX1* (intron.6)
AP736	RUNX1::RUNX1T1	*RUNX1* (intron.6)::*RUNX1T1* (intron.1); *RUNX1T1* (intron.1)::*RUNX1* (intron.6)
AP737	RUNX1::RUNX1T1	*RUNX1* (intron.6)::*RUNX1T1* (intron.1)
AP431	RUNX1::RUNX1T1	*RUNX1* (intron.6)::*RUNX1T1* (intron.1)
AP459	*PML*::*RARA* (L-type)	*PML* (intron.6)::*RARA* (intron.2)
AP478	*PML*::*RARA* (L-type)	*PML* (intron.6)::*RARA* (intron.2); *RARA* (intron.2)::*PML* (intron.6)
AP561	*PML*::*RARA* (L-type)	*PML* (intron.6)::*RARA* (intron.2); *RARA* (intron.2)::*PML* (intron.6)
AP604	*PML*::*RARA* (L-type)	*PML* (intron.6)::*RARA* (intron.2); *RARA* (intron.2)::*PML* (intron.6)
AP611	*PML*::*RARA* (L-type)	*PML* (intron.6)::*RARA* (intron.2); *RARA* (intron.2)::*PML* (intron.6)
AP626	*PML*::*RARA* (L-type)	*PML* (exon.7)::*RARA* (intron.2); *RARA* (intron.2)::*PML* (exon.7)
AP678	*PML*::*RARA* (L-type)	*PML* (intron.6)::*RARA* (intron.2); *RARA* (intron.2)::*PML* (intron.6)
AP772	*PML*::*RARA* (L-type)	*PML* (intron.6)::*RARA* (intron.2); *RARA* (intron.2)::*PML* (intron.6)
AP485	*PML*::*RARA* (S-type)	*PML* (intron.3)::*RARA* (intron.2); *PML* (intron.5)::*RARA* (intron.2)
AP514	*PML*::*RARA* (S-type)	*PML* (intron.3)::*RARA* (intron.2); *RARA* (intron.2)::*PML* (intron.3)
AP595	*PML*::*RARA* (S-type)	*PML* (intron.3)::*RARA* (intron.2)
AP636	*PML*::*RARA* (S-type)	*PML* (intron.3)::*RARA* (intron.2); *RARA* (intron.2)::*PML* (intron.3)
AP672	*PML*::*RARA* (S-type)	*PML* (intron.3)::*RARA* (intron.2); *RARA* (intron.2)::*PML* (intron.3)
AP726	*PML*::*RARA* (S-type)	*RARA* (intron.1)::*PML* (intron.6)
AP767	*PML*::*RARA* (S-type)	*PML* (intron.3)::*RARA* (intron.2)
AP858	*PML*::*RARA* (S-type)	*PML* (intron.3)::*RARA* (intron.2); *RARA* (intron.2)::*PML* (intron.7)
AP878	*PML*::*RARA* (S-type)	*PML* (intron.3)::*RARA* (intron.2); *RARA* (intron.2)::*PML* (intron.3)
AP609	*PML*:*RARA* (V-type)	*PML* (exon.6)::*RARA* (intron.2); *RARA* (intron.2)::*PML* (exon.6)
AP432	KMT2A::MLLT3	*KMT2A* (intron.10)::*MLLT3* (intron.5); *MLLT3* (intron.5)::*KMT2A* (intron.10)
AP466	KMT2A::MLLT3	*KMT2A* (intron.10)::*MLLT3* (intron.5); *MLLT3* (intron.5)::*KMT2A* (intron.10)
AP619	KMT2A::MLLT3	*KMT2A* (intron9)::*MLLT3* (exon.5); *MLLT3* (exon.5)::*KMT2A* (intron9)
AP691	KMT2A::MLLT3	*KMT2A* (exon.11)::*MLLT3* (intron.5); *MLLT3* (intron.5)::*KMT2A* (exon.11)
AP724	KMT2A::MLLT3	*KMT2A* (intron.10)::*MLLT3* (intron.5)
AP787	KMT2A::MLLT3	*KMT2A* (intron.10)::*MLLT3* (intron.5); *MLLT3* (intron.5)::*KMT2A* (intron.10)
AP528	Beyond the detection range of qRT-PCR	*KMT2A* (intron.10)::*MLLT3* (intron.5)
AP462	KMT2A::ELL	*KMT2A* (intron.10)::*ELL* (intron.1)
AP881	KMT2A::ELL	*KMT2A* (intron.10)::*ELL* (intron.1); *ELL* (intron.1)::*KMT2A* (intron.10)
AP440	Failed (The amplification of internal reference *ABL1* is not qualified)	*KMT2A* (intron.10)::*ELL* (intron.1)
AP603	KMT2A::MLLT1	*MLLT1* (intergenic)::*KMT2A* (intron.9)
AP608	KMT2A::MLLT1	*KMT2A* (intron.8)::*MLLT1* (intron.6); *MLLT1* (intron.6)::*KMT2A* (intron.8)
AP816	KMT2A::MLLT1	*MLLT1* (intergenic)::*KMT2A* (intron.10)
AP873	KMT2A::MLLT10	*KMT2A* (intron.8)::*MLLT1*0 (intron.2)
AP855	KMT2A::AFDN	*KMT2A* (intron.8)::*AFDN* (intron.1)
AP676	KMT2A::AFF1	*KMT2A* (intron.9)::AFF1 (intron.4)
AP760	DEK::NUP214	Negative
AC141	SET::NUP214	*SET* (exon.8)::*NUP214* (intron.16)
AP746	FUS::ERG	*FUS* (intron.7)::*ERG* (intron.9); *ERG* (intron.9)::*FUS* (intron.7)
AC366	NUP98::NSD1	*NUP98* (intron.12)::*NSD1* (intron.5); *NSD1* (intron.5)::*NUP98* (intron.12)
AP424	NUP98::HOXA9	*NUP98* (intron.12)::*HOXA9* (intron.1)
AP875	RUNX1::MECOM	*RUNX1* (intron.6)::*MECOM* (intron.1)
AP637	RUNX1::MECOM	Negative
AP474	TCF3::HLF	Negative
AP413	TCF3::PBX1	*TCF3* (intron.16)::*PBX1* (intron.2)

The tNGS identified 98 gene fusions, with the remaining 4 gene fusions not detected, which included one each of *CBFB*::*MYH11*, *DEK*::*NUP214*, *RUNX1*::*MECOM*, and *TCF3*::*HLF* (Fig 1, Table 2). tNGS on DNA-level can identify 96.1% (98/102) (positive percent agreement, PPA) of the gene fusions previously detected by qRT-PCR on RNA-level (Fig 1). The tNGS method was unable to successfully detect fusion genes in four samples. In the intron regions corresponding to the *CBFB*, *NUP214*, *RUNX1*, and *TCF3* genes, there were no significant differences observed in the proportion of low-coverage regions and the overall assessment values. Intron 4 and 5 of the *CBFB* gene contained 19.8% of low-coverage regions compared to an average of 21.1%; intron 17 of the *NUP214* gene contained 45.6% of low-coverage regions compared to an average of 50.6%; intron 5 of the *RUNX1* gene contained 4.3% of low-coverage regions compared to 2.7%; and intron 15 and 16 of the *TCF3* gene contained 8.8% of low-coverage regions compared to 6.7%. We hypothesize that the breakpoints may be situated precisely within the low-coverage regions, which explains their undetection, as observed in cases such as *CBFB*::*MYH11* and *DEK*::*NUP214*. Additionally, it is possible that the breakpoints fall outside the coverage range of the panel, contributing to their lack of detection such as *RUNX1*::*MECOM* and *TCF3*::*HLF*.

**Fig 2:**
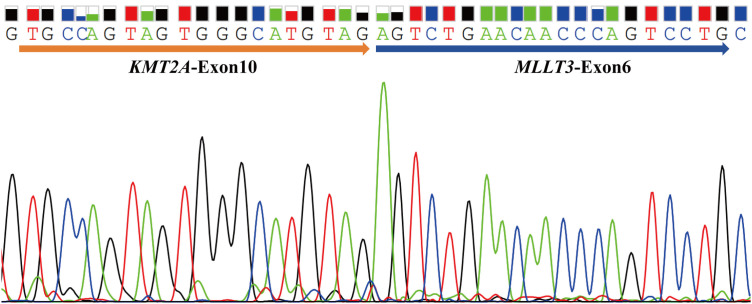
Validation of the *KMT2A*::*MLLT3* (exon 10::exon 6) gene fusion by Sanger sequencing. Sample AP528 harbored a rare fusion gene, *KMT2A*::*MLLT3* (exon 10::exon 6), identified using NGS methodology, exceeding the detection capabilities of qRT-PCR. Consequently, primers were devised for amplifying the fusion gene at the RNA level and validated via Sanger sequencing.

The tNGS analysis also identified two gene fusions, *KMT2A*::*ELL* and *KMT2A*::*MLLT3*, that had not been detected through the qRT-PCR method (Table 2). Theoretically, tNGS detects gene fusions on DNA-level with an negative percent agreement (NPA) of 99.2% (241/243) compared to the detection results on RNA-level using qRT-PCR. Following the analysis, it was discovered that *KMT2A*::*MLLT3* exhibits an uncommon fusion pattern (exon 10::exon 6) which fell outside the detection capability of qRT-PCR. This fusion gene was subsequently amplified on RNA-level with specifically designed primers and confirmed by Sanger sequencing (Fig 2). The sample labeled as AP528 did not amplify the internal control gene *ABL1* during qRT-PCR analysis, resulting in the absence of the detection of the fusion gene *KMT2A*::*ELL*. During cytogenetic analysis at the time of diagnosis, the t(11;19)(q23.3;p13.1) chromosomal translocation was identified in the AP528 samples, thereby confirming the authenticity of the fusion genes detected through tNGS (S4 Table).

### Analysis of special results from tNGS testing

A significant proportion of 55% (55/100) in gene fusion-positive samples exhibited the presence of both primary and reciprocal gene fusions upon undergoing tNGS testing. For instance, the detection of *BCR*::*ABL1* and *ABL1*::*BCR* gene fusions occurred simultaneously. The definition of “reciprocal gene fusion” is the juxtaposition of the 5’ portion of a kinase gene and the 3’ portion of a partner gene [39]. Among the remaining 45% (45/100) of samples, only one fusion pattern is observed in the gene fusions.

Of particular note is that in tNGS detection, 5 samples only showed reciprocal gene fusions, comprising 2 cases of *MLLT1*::*KMT2A*, one case each of *MYH11*::*CBFB*, *ABL1*::*BCR*, and *RARA*::*PML*. Meanwhile, the qRT-PCR testing on RNA-level conducted simultaneously proved that these samples coexist with primary gene fusions. This implies that on DNA-level detection of gene fusions using tNGS, if only reciprocal fusions are detected, further assessment on RNA-level is warranted.

Sample AP485 was identified with two gene fusions through tNGS analysis: *PML*::*RARA* (exon 3::exon 3, S-type) and *PML*::*RARA* (exon 5::exon 3, atypical), revealing 195 and 69 reads for the respective gene fusions (S1 Fig). RNA sample was utilized for PCR amplification and Sanger sequencing of these two gene fusions. Ultimately, only the *PML*::*RARA* (exon 3::exon 3, S-type) transcript was identified, while the atypical transcript of *PML::RARA* (exon 5::exon 3) remained undetected. Earlier studies have indicated that certain rearrangements at the DNA level do not result in the formation of fusion transcripts at the RNA level [39]. It is therefore inferred that the cases presented in this study may exhibit a similar phenomenon.

### Chromosomal alterations in karyotype analysis

Of the 104 samples in which gene fusions were detected through qRT-PCR or NGS, 58 exhibited results from chromosomal karyotype analysis (S4 Table). Only two samples did not exhibit genetic abnormalities associated with the gene fusions, both of which corresponded to the *KMT2A*::*MLLT1* gene fusion. Because *KMT2A* and *MLLT1* are located at the ends of chromosomes, this translocation is nearly cryptic, making it easy for traditional chromosomal karyotype analysis to miss or fail to detect. For cryptic translocations, molecular techniques such as qRT-PCR or tNGS are more effective detection methods, as they can bypass the limitations of chromosomal karyotype analysis.

### *IGH* and *MYC* rearrangement detected by tNGS

Among the 76 samples diagnosed with B-ALL, **IGH,* IGL* or *MYC* rearrangement was identified in six samples through tNGS. Three cases exhibited *IGH*::*MYC* rearrangements, one case exhibited an *MYC*::*GRHPR* rearrangement, and one case exhibited an *IGH*::*CRLF2* rearrangement. Notably, one case of patient with *IGL*::*MYC* rearrangement was also detected. All 76 B-ALL patients underwent FISH detection for *IGH* rearrangement, and only four samples tested positive for *IGH* rearrangement. Additionally, these four samples were found to have *IGH*::*CRLF2* or *IGH*::*MYC* detected by tNGS. The patient identified with the **I*GL*::*MYC* and *MYC*::*GRHPR* rearrangement subsequently underwent FISH testing using *MYC* dual-color separation probes, which yielded positive results for *MYC* rearrangement (S2 Fig).

To adequately demonstrate the utility of the leukemia tNGS panel in detecting gene rearrangements, including *IGH* and *MYC*, in patients with B-ALL, we selected an additional ten B-ALL samples that were positive for the *IGH* dual-color separation probe by FISH and subjected them to analysis using the leukemia tNGS panel. Among the ten samples, nine exhibited *IGH* gene rearrangements, including six cases of *IGH*::*MYC* and one case each of *IGH*::*EPOR*, *IGH*::*USP7*, and *IGH*::*ARID1B* (Table 3). It is speculated that the lack of detection of the rearrangement may be attributed to the breakpoints not falling within the detection range of this panel. The final sample, which did not exhibit *IGH* rearrangement, was not subjected to whole genome sequencing due to cost constraints.

**Table 3:** *IGH* or *MYC* rearrangements detected by FISH or tNGS in B-ALL patients.

Sample number	Diagnosis	Results OF FISH^a^	tNGS detection result		
			Gene rearrangement	Left breakpoint	Right breakpoint
AR703	B-ALL	FISH: *IGH* (+)	CRLF2::IGH	chrX:1357410	chr14:106329450
AR515	B-ALL	FISH: *MYC* (+)	IGL::MYC	chr22:23248512	chr8:128754255
AR458	B-ALL	FISH: *IGH* (+)	IGH::MYC	chr14:106329450	chr8:128748142
AR423	B-ALL	FISH: *IGH* (+)	IGH::MYC	chr14:106326156	chr8:128748986
LP2730	B-ALL	FISH: *IGH* (+)	IGH::MYC	chr14:106189137	chr8:128749490
LP1859	B-ALL	FISH: *MYC* (+)	MYC::GRHPR	chr8:128755172	chr9:37400679
AP1287	B-ALL	FISH: *IGH* (+)	EPOR::IGH	chr19:11488866	chr14:106725200
			IGH::EPOR	chr14:106329454	chr19:11498050
LP2428	B-ALL	FISH: *IGH* (+)	IGH::USP7	chr14:107337388	chr16:8988946
LS340	B-ALL	FISH: *IGH* (+)	ARID1B::IGH	chr6:157099234	chr14:106032901
ASP24061101	B-ALL	FISH: *IGH* (+)	IGH::MYC	chr14:106176950	chr8:128749432
ASP24101101	B-ALL	FISH: *IGH* (+)	MYC::IGH	chr8:128746169	chr14:106324828
			MYC::IGH	chr8:128746107	chr14:106325014
ASP25031701	B-ALL	FISH: *IGH* (+)	MYC::IGH	chr8:128749265	chr14:106325405
ASP24100801	B-ALL	FISH: *IGH* (+)	IGH::MYC	chr14:106239941	chr8:128748258
LP1991	B-ALL	FISH: *IGH* (+)	MYC::IGH	chr8:128748879	chr14:106055830
			IGH::MYC	chr14:106055810	chr8:128748878
LP1764	B-ALL	FISH: *IGH* (+)	MYC::IGH	chr8:128749054	chr14:106049908
			IGH::MYC	chr14:106049912	chr8:128749060
LP1355	B-ALL	FISH: *IGH* (+)	undetected		

^a^The FISH detection results for the *IGH* and *MYC* genes were both performed using dual-color separation probes.

The detection of *IGH* or *MYC* rearrangements using tNGS in patients with B-ALL achieved a PPA of 93.8% (15/16) with FISH results. Furthermore, tNGS can accurately identify the specific partner genes associated with these rearrangements, facilitating a more precise analysis of the impact of mutations on prognosis.

## Discussion

Adult patients diagnosed with leukemia, including AML and ALL, exhibit a spectrum of genetic alterations that are increasingly utilized for refine the prognosis and guiding individualized treatment strategies. These genetic variations encompass single nucleotide substitutions, small insertions and deletions (indels), as well as changes that involve large genomic regions, such as translocations, inversions, chromosomal duplications and deletions [4]. Established guidelines advocate for the application of conventional karyotype analysis, FISH, and/or qRT-PCR assays to identify genomic rearrangements [3]. Nonetheless, these customary analyses represent labor-intensive procedures that impede the efficiency of diagnostic workflows. The latest advancements in molecular technologies, such as tNGS, have found extensive application in the diagnosis of leukemia. Presently, targeted genome sequencing stands as the predominant method utilized, exhibiting the capacity to concurrently detect tens or even hundreds of gene mutations with heightened sensitivity, while also considering the cost-effective aspect of detection [3]. Nevertheless, targeted sequencing for leukemia predominantly concentrates on identifying single nucleotide substitutions and indels, necessitating the assistance of qRT-PCR for the additional detection of gene fusions. Consequently, novel and unidentified genomic rearrangements remain undetectable.

Utilizing DNA for simultaneous detection of mutations and gene fusions, this tNGS strategy relies solely on DNA samples, eliminating the requirement of RNA, thereby streamlining laboratory procedures and enhancing quality control measures. Moreover, it is capable of detecting numerous previously unidentified genomic rearrangements, which, upon discovery, could significantly influence clinical decision-making for patients who might otherwise be erroneously classified as having a normal karyotype [40]. Another notable benefit of this leukemia tNGS panel is its requirement of a minimal DNA quantity (50 ng) to effectively screen a wide range of significant genomic rearrangements in leukemia.

Several studies have confirmed the viability of employing tNGS on DNA-level to identify gene fusions in hematologic malignancies. However, these studies are restricted to detecting a limited number of gene fusions in AML patients or have a small sample size [41–43], which hinders the ability to fully validate the accuracy and limitations of this approach. This study represents the most extensive research to date utilizing tNGS on DNA-level for the detection of gene fusions in hematologic malignancies. This study does not solely focus on myeloid tumors, it also encompasses a cohort of patients diagnosed with ALL. The leukemia panel encompasses 94 introns from 26 genes, making it the panel that covers the most introns to date and is capable of detecting dozens gene fusions [41,42]. This study has shown the feasibility of detecting gene fusions on DNA-level using tNGS methods. This method not only reduces the amount of samples required but also minimizes the labor requirement. Furthermore, tNGS has the capability to identify novel and unrecognized gene fusions. Nonetheless, for atypical gene fusions identified through tNGS methods, additional validation on RNA-level is essential to ensure the accuracy of the detection outcomes.

When employing tNGS for the identification of gene fusions on DNA-level, it is possible to encounter cases where only the reciprocal fusion gene is identified [approximately 1.4% (5/357) of the total sample in this study], such as the *RARA*::*PML*. The clinical implications of this reciprocal fusion gene in comparison to typical gene fusions will impact patients’ treatment decisions. At this stage, additional methods are required for RNA-level validation, including techniques like qRT-PCR, Sanger sequencing and RNA-seq. In this study, all five cases showing only reciprocal gene fusions were confirmed by qRT-PCR to display traditional primary gene fusions on RNA-level. Due to the complexity of DNA-level rearrangements, earlier studies on the detection of gene fusions have identified three scenarios: the simultaneous presence of primary and reciprocal rearrangements, instances with only reciprocal rearrangements, and those containing only primary rearrangements [39,43]. Furthermore, in these studies, cases that identified only reciprocal rearrangements predominantly confirmed the presence of primary rearrangements through RT-PCR. The mechanisms underlying the formation of these processes remain unclear; however, on DNA-level, atypical fusion genes can result in the production of oncogenic gene fusion transcripts in over 80% of cases [44]. This is a significant limitation of tNGS in the detection of gene fusions at the DNA level. The identification of atypical gene fusions on DNA-level using tNGS, such as reciprocal gene fusions, uncommon fusion types, or rare fusion partner genes, requires further validation on RNA-level to ensure result accuracy.

Genomic rearrangements on DNA-level have the potential to give rise to a diverse array of gene fusions [14]. An intergenic breakpoint can lead to the upregulation of a gene through the action of an alternate promoter or enhancer that has been relocated upstream of the gene. This phenomenon is commonly described as promoter or enhancer “swapping” or “hijacking”, a illustration is the translocation event involving proto-oncogenes and *IGH* region in lymphomas [45]. Utilizing tNGS on DNA-level allows for the detection of these genomic rearrangements [16,46], which is a significant advantage of this methodology. The consistency of results between tNGS and FISH for detecting *MYC*/*BCL2*/*BCL6* rearrangements in lymphoma ranged from approximately 80% to 90% [13,16,47]. In this study, the PPA for detecting *IGH* or *MYC* rearrangements in B-ALL samples using tNGS and FISH reached 93.8%, which is consistent with or slightly higher than reported in the literature. Concurrently, we observed that rearrangements discovered in this study such as *MYC*::*GRHPR*, *IGH*::*USP7*, and *IGH*::*ARID1B* represent atypical forms of rearrangement, and their impact on prognosis may be minimal [17]. FISH methods are unable to discern the specific types of rearrangements, potentially leading to erroneous clinical interpretations. The tNGS can identify specific rearrangement breakpoints and partner genes, thereby enhancing the accuracy of its clinical implications.

The leukemia tNGS panel can detect fusion genes on DNA-level of leukemia patients with high sensitivity and specificity. Its implementation in clinical settings has the potential to significantly reduce the workload of laboratory technicians and decrease testing costs for patients.

## Supporting information

S1 Table A list of 302 genes that encompass all exons. (XLSX)

S2 Table 45 fusion genes and fusion types that could be detected by qRT-PCR. (XLSX)

S3 Table Genomic coordinates, lengths, and percentages of regions with low sequencing depth. (XLSX)

S4 Table The similarities and differences between the fusion gene results detected by qRT-PCR and tNGS methods, as well as the results of karyotype analysis. (XLSX)

S1 Fig The two types of *PML*::*RARA* gene fusions detected in the AP485 sample. Sample AP485 was identified with two fusions through tNGS analysis: *PML*::*RARA* (exon 3::exon 3, S-type) and *PML*::*RARA* (exon 5::exon 3, atypical). Figure A and B represent reads of S-type gene fusions, while figures C and D represent reads of atypical gene fusions.
(PDF)

S2 Fig The images of ALL patients exhibiting positive results for IGH or MYC gene rearrangement during FISH testing are presented. Figures A-C present the results of FISH analysis using *IGH* gene dual-color separation probes for three patients who tested positive for *IGH*::*MYC* rearrangement. Figure D illustrates the FISH detection results of *IGH* gene dual-color separation probes for a sample positive for *IGH*::*CRLF2* rearrangement. Figure E presents the FISH detection results of *MYC* gene dual-color separation probes for a sample positive for *IGL*::*MYC* rearrangement. Figure F presents the FISH detection results of *MYC* gene dual-color separation probes for a sample positive for *MYC*::*GRHPR* rearrangement.
(PDF)
