# Supplementary material for: Detection of leukemia gene fusions on DNA-level through targeted Next-Generation Sequencing
Source: PLoS One. 2025 Oct 9;20(10):e0332407. doi: 10.1371/journal.pone.0332407 (PMC12510534; doi:10.1371/journal.pone.0332407)

S1 Fig. The two types of *PML*::*RARA* gene fusions detected in the AP485 sample. Sample AP485 was identified with two fusions through tNGS analysis: *PML*::*RARA* (exon 3::exon 3, S-type) and *PML*::*RARA* (exon 5::exon 3, atypical). Figure A and B represent reads of S-type gene fusions, while figures C and D represent reads of atypical gene fusions.

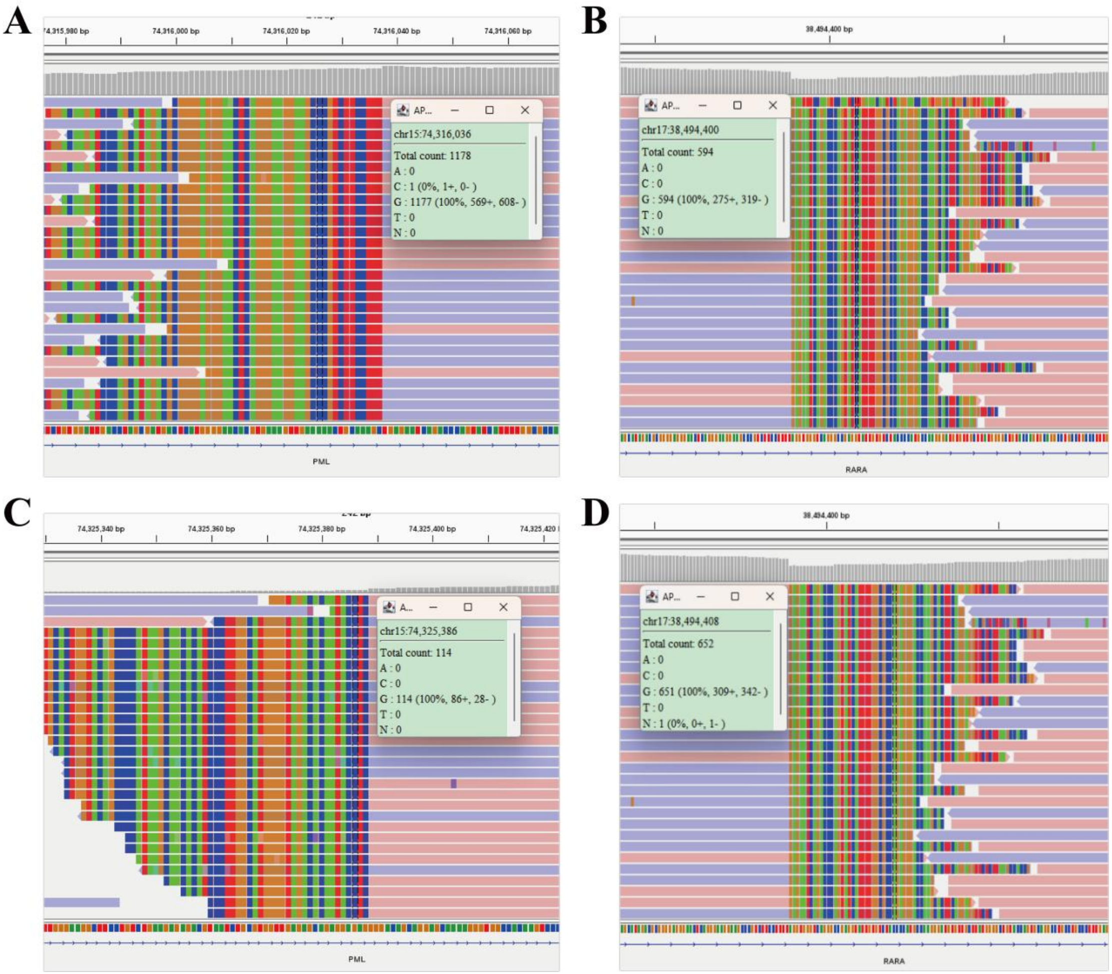

Supplement: S1 Fig — Sample AP485 was identified with two fusions through tNGS analysis: PML::RARA (exon 3::exon 3, S-type) and PML::RARA (exon 5::exon 3, atypical). Figure A and B represent reads of S-type gene fusions, while figures C and D represent reads of atypical gene fusions. (PDF) [file pone.0332407.s005.pdf]
