# Supplementary material for: Detection of leukemia gene fusions on DNA-level through targeted Next-Generation Sequencing
Source: PLoS One. 2025 Oct 9;20(10):e0332407. doi: 10.1371/journal.pone.0332407 (PMC12510534; doi:10.1371/journal.pone.0332407)
Supplement: S2 Fig — Figures A-C present the results of FISH analysis using IGH gene dual-color separation probes for three patients who tested positive for IGH::MYC rearrangement. Figure D illustrates the FISH detection results of IGH gene dual-color separation probes for a sample positive for IGH::CRLF2 rearrangement. Figure E presents the FISH detection results of MYC gene dual-color separation probes for a sample positive for IGL::MYC rearrangement. Figure F presents the FISH detection results of MYC gene dual-color separation probes for a sample positive for MYC::GRHPR rearrangement. (PDF) [file pone.0332407.s006.pdf]

S2 Fig. The images of ALL patients exhibiting positive results for *IGH* or *MYC* gene rearrangement during FISH testing are presented. Figures A-C present the results of FISH analysis using IGH gene dual-color separation probes for three patients who tested positive for IGH::MYC rearrangement. Figure D illustrates the FISH detection results of IGH gene dual-color separation probes for a sample positive for IGH::CRLF2 rearrangement. Figure E presents the FISH detection results of MYC gene dual-color separation probes for a sample positive for IGL::MYC rearrangement. Figure F presents the FISH detection results of MYC gene dual-color separation probes for a sample positive for MYC::GRHPR rearrangement.

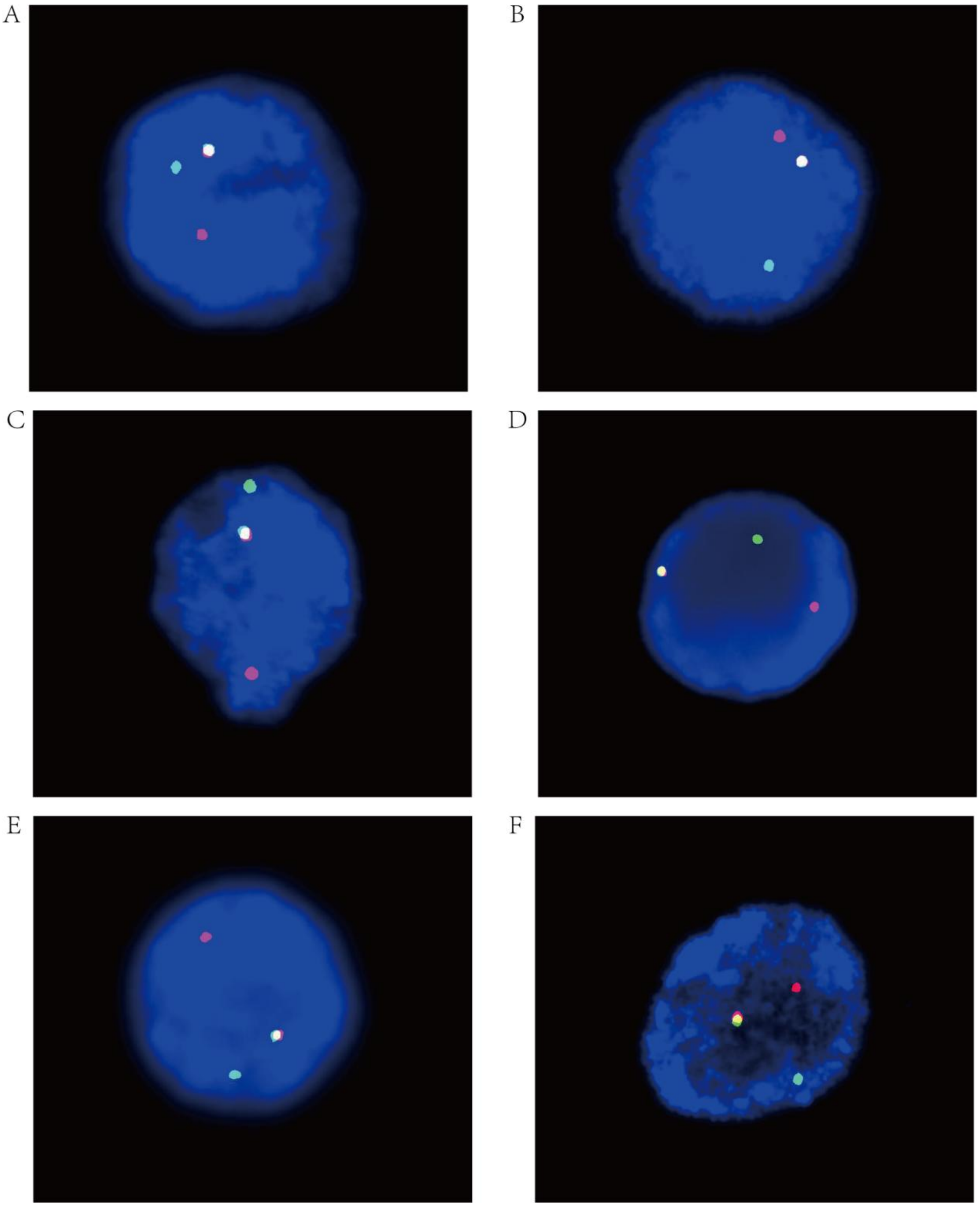
A
B
C
D
E
F
